# Paltusotine, a novel oral once-daily nonpeptide SST2 receptor agonist, suppresses GH and IGF-1 in healthy volunteers

**DOI:** 10.1007/s11102-021-01201-z

**Published:** 2022-01-09

**Authors:** Ajay Madan, Stacy Markison, Stephen F. Betz, Alan Krasner, Rosa Luo, Theresa Jochelson, Jason Lickliter, R. Scott Struthers

**Affiliations:** 1 Crinetics Pharmaceuticals, Inc, 10222 Barnes Canyon Road, Building 2, San Diego, CA 92121 USA; 2 Nucleus Network, Melbourne, VIC 3004 Australia; 3 Crinetics Medical Affairs, Crinetics Pharmaceuticals, Inc, 10222 Barnes Canyon Rd. Bldg.2, San Diego, CA 92121 USA

**Keywords:** Paltusotine, Acromegaly, IGF-1, SST2, Somatostatin receptor agonist

## Abstract

**Purpose:**

Evaluate the pharmacodynamics, pharmacokinetics, and safety of paltusotine, an orally bioavailable, nonpeptide, somatostatin receptor subtype 2 (SST2) agonist being developed for the treatment of acromegaly and neuroendocrine tumors.

**Methods:**

A randomized, double-blind, placebo-controlled, single center, single and multiple ascending dose phase 1 study was conducted in healthy male volunteers who received (i) single-dose of oral paltusotine 1.25, 2.5, 5, 10, and 20 mg (solution); and 40 and 60 mg (capsules) or (ii) multiple-dose oral paltusotine capsules once daily 5 mg (× 7 days), 10, 20, and 30 mg (× 10 days). Main outcome measures were pharmacodynamics (changes in growth hormone-releasing hormone [GHRH] stimulated growth hormone [GH] and insulin-like growth factor 1 [IGF-1]), pharmacokinetics, safety, and tolerability.

**Results:**

Single-dose cohorts: n = 41 active, n = 14 placebo. Multiple-dose cohorts: n = 24 active, n = 12 placebo. Paltusotine was well tolerated, orally bioavailable, associated with increased plasma concentrations to doses up to 40 mg, and was eliminated with a half-life of approximately 30 h. Single-dose paltusotine 1.25 to 20 mg suppressed GHRH-stimulated GH secretion by 44% to 93% compared to 15% with placebo. Multiple-dose paltusotine 5 to 30 mg administered once daily for 10 days suppressed IGF-1 by 19% to 37% compared to an increase of 2.4% with placebo.

**Conclusions:**

Paltusotine suppresses GH and IGF-1 in a dose-dependent fashion, with a safety profile similar to currently approved SST2 receptor ligands. Paltusotine is a promising once-daily oral nonpeptide SST2 agonist candidate for managing acromegaly and neuroendocrine tumors.

**Trial registration:**

NCT03276858, registered September 8, 2017, retrospectively registered.

## Background

Somatostatin is produced by a variety of cell types in the central nervous system and gut and has pleiotropic effects, many of which are related to inhibiting the secretion of other hormones. The physiologic and pharmacologic activity of somatostatin is mediated by five somatostatin receptors (SST1, SST2, SST3, SST4, SST5) [1, 2] that are selectively expressed in different tissues. The type 2 receptor, SST2, is expressed in pituitary somatotrophic cells where it mediates the inhibition of growth hormone (GH) secretion, which in turn causes secretion of insulin-like growth factor 1 (IGF-1) from the liver and is also highly expressed in gut enteroendocrine and pancreatic islet cells, as well as neuroendocrine tumors that arise from these tissues [3]. Synthetic peptide SST2 receptor ligands, such as octreotide and lanreotide, inhibit secretory activity of somatotrophs and gastroenteropancreatic neuroendocrine tumors (GEPNETs) and also improve progression free survival in patients with GEPNETs [4, 5]. Pasireotide, a synthetic peptide pan somatostatin receptor ligand (SRL) with activity at SST2, SST3, and SST5 receptors is also approved for acromegaly. The efficacy of pasireotide in acromegaly is in part attributed to its relative potent activity at SST5, which is also causally associated with hyperglycemia/diabetes in approximately 57% of the patients taking it [6]. The clinical use of these drugs allows patients diagnosed with acromegaly to have an increased life expectancy and improves progression free survival in GEPNETs; however, debilitating symptoms and sequelae from their conditions often remain [7, 8]. These SRLs are peptide drugs and are most frequently administered by healthcare professionals as once-monthly depot injections. In addition to the inconvenience of scheduling injections [8–10], successful delivery of the injections are operator-dependent, [11], and approximately half of acromegaly patients on these drugs experience a return of symptoms at the end of the monthly cycle [8]. More recently, a twice-daily oral preparation of octreotide has become available which can maintain IGF-1 control in 58% of patients who were previously controlled on depot preparations [12].

To overcome some of the limitations of peptide somatostatin ligands, an iterative medicinal chemistry approach was employed to discover a first-in-class, nonpeptide, orally available small molecule somatostatin agonist highly selective for SST2. These efforts led to the selection of paltusotine (formerly CRN00808) for preclinical and clinical development as a potential treatment of acromegaly and GEPNETs. Paltusotine is a highly potent SST2 agonist (EC_50_ = 0.25 nM); is > 4000-fold selective for SST2 over other somatostatin receptor subtypes; suppresses stimulated-GH and IGF-1 secretion in rats [13]; and has a high oral bioavailability in animals [14]. Here we report results from a first-in-human phase 1 study to evaluate the pharmacological activity (stimulated-GH and IGF-1 suppression), pharmacokinetics, safety, and tolerability of paltusotine after single- and multiple-dose administration in healthy volunteers.

## Methods and research participants

### Study protocol

A phase 1, first-in-human, randomized, double-blind, placebo-controlled, single and multiple ascending dose study was performed with oral paltusotine at a single study center in Melbourne, Australia. Inclusion criteria included age 18 to 50 years; body mass index of 18–30 kg/m^2^; and if female, postmenopausal by history or surgery. Exclusion criteria included presence of significant medical history. Participants had normal laboratory tests, including hematology and chemistry panels and liver enzymes, blood pressure, and electrocardiogram (ECG) at screening. The trial was registered at www.clinicaltrials.gov (NCT03276858) and was conducted in accordance with Good Clinical Practice and the principles in the Declaration of Helsinki. The study was approved by the Alfred Hospital Ethics Committee (Melbourne, Australia). Participants provided written informed consent before any study procedures.

Screening occurred within 28 days prior to the first administered dose (Day 1) in both single-dose and multiple-dose cohorts. Participants were confined to a clinical research unit from Day -2 until the day after the last dose. Dosing of paltusotine or placebo commenced on Day 1 after baseline studies. Participants fasted overnight for ≥ 10 h prior to each dose administration and remained fasting until 4 h post-dose (after collection of the last GH sample after growth hormone-releasing hormone [GHRH] challenge). For the food effect cohort, the study drug was administered 30 min after a high-fat and high-calorie breakfast. Crinetics Pharmaceuticals, Inc (San Diego, CA) provided the active ingredient, paltusotine (3-[4-(4-Aminopiperidin-1-yl)-3-(3,5-difluorophenyl)quinolin-6-yl]-2-hydroxybenzonitrile hydrochloride; CRN00808), for administration as either an oral solution (prepared on site at the Phase 1 unit) or as capsules (placebo, 5 mg, and 20 mg).

**Fig. 1:**
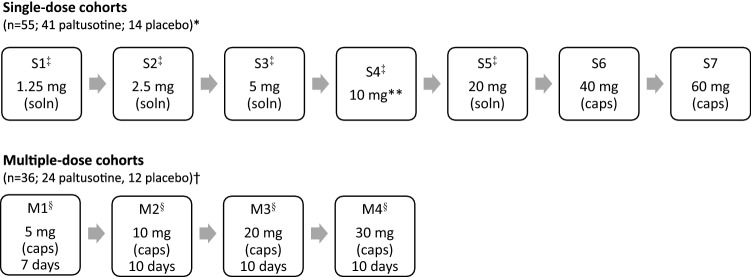
Trial design for single- and multiple-dose cohorts. Dose escalation in a new cohort, *soln* solution, *caps* capsule. *n = 8 per single-dose cohort (6 active, 2 placebo), except for cohort S5 in which one participant withdrew prior to dosing and was not replaced (n = 7; 5 active, 2 placebo). †n = 9 per multiple-dose cohort (6 active, 3 placebo). **There were 3 study periods in cohort S4: period A (10 mg solution); period B (10 mg capsules) and period C (10 mg capsules with food). ^‡^Single-dose pharmacodynamic population (n = 39; paltusotine n = 29; placebo n = 10) included participants in S1, S2, S3, S4A, and S5 cohorts. GHRH challenge was administered on Day 1 and 2 h post-dose on Day 1. ^§^Multiple-dose pharmacodynamic population (n = 31; paltusotine n = 21; placebo n = 10) included participants from the M1, M2, M3, and M4 cohorts who received all doses

Participants received oral paltusotine or placebo according to the scheme shown in Fig. 1. In the single-dose phase, participants were randomized 6:2 to paltusotine (1.25, 2.5, 5, 10, 20, 40, and 60 mg) or placebo in 7 cohorts (S1 to S7). Cohorts comparing oral bioavailability of solution versus capsules and the effect of a standardized high-fat, high-calorie breakfast on oral bioavailability of the capsule formulation employed the 10 mg dose level. In the multiple-dose phase, participants were randomized 6:3 to once-daily oral paltusotine capsules or placebo at doses of 5 mg for 7 days (M1, Day Last = Day 7), and 10, 20, and 30 mg daily for 10 days (M2, M3, M4, Day Last = Day 10). The initial starting dose of 1.25 mg in the single-dose phase was selected for this study based on nonclinical pharmacologic activity, pharmacokinetics, and safety studies. Subsequent doses were either specified in the protocol or were selected by the safety review committee responsible for the safety oversight of the study.

### Pharmacokinetic sampling

Single-dose cohorts: Plasma samples were collected on Day 1 (pre-dose, every 15 min up to 90 min post-dose, then at 2, 3, 4, 6, 8, 10, 12, 18, 24, 48, 72, 96, 120, and 144 h post-dose). Multiple-dose cohorts: For the 5 mg cohort, plasma samples were collected on Day 1 (pre-dose, every 15 min up to 90 min post-dose, then at 2, 3, 4, 6, 8, 10, 12, and 18 h post-dose), Days 2–6 (pre-dose and 2 h post-dose), Day 7 (same time points as Day 1, followed by samples at 18, 24, 48, 72, 96, 120, and 144 h post-dose). For all other multiple-dose cohorts, plasma samples were collected on Day 1 (pre-dose, every 15 min up to 90 min post-dose, then at 2, 3, 4, 6, 8, 10, 12, and 18 h post-dose), Days 2–6 (pre-dose and 2 h post-dose), Day 10 (same time points as Day 1, followed by samples at 18, 24, 48, 72, 96, 120, 144, 192, and 240 h post-dose). The last time point at which blood sample was collected for pharmacokinetics and terminal elimination half-life (t1/2) determination was 144 h post-dose for single-dose cohorts and cohort M1 and 240 h post-dose for cohorts M2-M4.

### Assessment of GH and IGF-1

To assess the ability of paltusotine to suppress GHRH-stimulated GH secretion, a GHRH challenge was performed in single-dose cohorts S1, S2, S3, S4, and S5 (oral solution) (Fig. 1). Recombinant human GHRH(1-44)-NH_2_ (somatorelin; Ferring Pharmaceuticals, Parsippany, NJ) 50 µg was administered intravenously 2 h after paltusotine (or placebo) on Day 1 and at approximately the same time on Day -1 (± 10 min). A blood sample for serum GH was collected at − 60 min, − 30 min, immediately before GHRH challenge, and at 15, 30, 45, 60, 90, and 120 min post-challenge. Serum GH was also assessed in the M1 cohort. Serum GH sample was collected on Day - 1, on Days 1 and 7 (pre-dose, 0.5, 1, 2, 3, 4, 6, 8, 10, 12 and 18 h post-dose), Days 2–6 (pre-dose and 12 h post-dose), and Days 8, 10 and 14 (at approximately the same time as dosing).

For assessment of serum IGF-1, in the single-dose cohorts, serum IGF-1 was collected on Day - 1 and Day 1 (pre-dose to 18 h post-dose), and Day 2 and 7 (at approximately the same time GHRH was administered on Day 1). In the M1 cohort, IGF-1 samples were obtained pre-dose, 6 and 12 h post-dose on Days - 1, 1 and 7; pre-dose and 12 h post-dose on Days 2–6; and at approximately the same time as dosing on Days 8, 10, and 14. For M2 and subsequent cohorts, IGF-1 samples were obtained pre-dose, 6 and 12 h post-dose on Days - 1, 1 and 10; pre-dose and 12 h post-dose on Days 2–9; and at approximately the same time as dosing on Days 11, 14, and 21.

### Safety assessments

Safety assessments included monitoring of clinical laboratory tests, cardiovascular safety monitoring (continuous Holter monitoring, ECG, telemetry), and physical examinations conducted at scheduled times throughout the study in the research units as well as post-discharge (up to 9 days after the single-dose, or up to 11 days after the last dose for multiple-dose cohorts). Adverse events and concomitant medications were recorded. Laboratory samples for clinical chemistry, hematology, coagulation tests, and urinalysis were collected under fasted conditions. Participants who discontinued prematurely attended an early termination visit.

In the multiple-dose cohorts, baseline serum TSH, ACTH, and prolactin samples were collected on Day -2, and an oral glucose tolerance test (OGTT) was conducted on Day -7 or earlier. These assessments were repeated on Day 8 (M1) or Day 11 (M2 and subsequent cohorts). A safety review committee oversaw the study and made decisions to escalate, expand or decrease the dose for the cohorts based on a review of demographics, pharmacokinetics, pharmacodynamics, and safety data in a blinded fashion.

### Analytical methods

Serum was isolated, stored, and analyzed for IGF-1 and GH according to the instructions from the manufacturer (IGF-1 and human GH diagnostic assay, Siemens Immulite® 1000 systems).

Plasma pharmacokinetic sample analysis for paltusotine was determined using validated procedures by CPR Pharma Services (Thebarton, Australia). Briefly, plasma paltusotine concentrations were determined by high-pressure liquid chromatography (HPLC) coupled with tandem mass spectrometry detection (MS/MS). Deuterated paltusotine was used as an internal standard. Supported liquid extraction was used to extract paltusotine and the internal standard. The analyte was separated by HPLC using an ACE C18-AR column (Advanced Chromatography Technologies, Ltd, Aberdeen, Scotland), and the eluates were monitored by an API 4000™ LC–MS/MS System (Sciex, Framingham, MA) in positive multiple reaction monitoring mode. The extract was then assayed against a calibration curve. The data were acquired and integrated by the data acquisition software Analyst® (Sciex), linked directly to the API 4000™ LC–MS/MS System, and then processed using Watson LIMS™ software (Thermo Scientific, Waltham, MA), where applicable. The calibration range was 0.100 to 100 ng/mL using 100 μL of plasma, with a lower limit of quantification of 0.100 ng/mL. The inter-assay accuracy was 94% to 100%, and the inter-assay precision (% CV) was between 6.1% and 15%.

### Statistical analysis

The study endpoints were effects of paltusotine on GHRH-stimulated GH and IGF-1 (pharmacodynamics), pharmacokinetics (including formulation and food effects), safety, and tolerability. For the analysis of effects paltusotine on GHRH-stimulated GH and IGF-1, the pharmacodynamic population included all randomized participants in the 1.25 mg, 2.5 mg, 5 mg, 10 mg, and 20 mg (S1, S2, S3, S4A, and S5) single-dose cohorts who received any amount of study drug and had at least one post-baseline pharmacodynamic assessment. The percent change in serum GH area under the curve (AUC) from 0 to 2 h (AUC_0-2_) from baseline was compared using ANOVA between each dosing cohort and all participants who received placebo. The single-dose pharmacodynamic population included 39 participants from the S1, S2, S3, S4, and S5 cohorts (paltusotine n = 29; placebo n = 10). One participant each in the M1, M3, and M4 cohorts, and two placebo-treated participants did not meet the criteria for inclusion in the pharmacodynamic population because they did not receive all doses. For multiple-dose cohorts, only participants who received all doses were included. A change in IGF-1 levels 12 h post last dose from baseline (average of 3–4 measurements) were compared by ANOVA between each dosing cohort and all participants who received placebo. The multiple-dose pharmacodynamic population included 31 participants from the M1, M2, M3, and M4 cohorts (paltusotine n = 21; placebo n = 10).

For assessing pharmacokinetics of paltusotine, the single-dose pharmacokinetic population included all randomized participants who received any amount of study drug with sufficient plasma concentration data. For the multiple-dose cohorts, only participants who received all doses were included. The relative bioavailability of the capsule formulation was determined by comparing its pharmacokinetic parameters with those of the solution formulation at the 10 mg dose. Food effect was determined at the same dose by comparing pharmacokinetic parameters in the fasting state to those when paltusotine was administered with a high-fat, high-calorie meal. For both formulation and food effect, ANOVA with treatment as a fixed effect and subject as a random effect was performed. Data for peak concentration (Cmax), AUC0-last, and AUC0-inf were natural log-transformed.

For the safety analysis, adverse events were coded using the Medical Dictionary for Regulatory Activities (MedDRA® Version 20.0). Adverse events were grouped by preferred terms. Treatment-emergent adverse events (TEAEs) were summarized with descriptive statistics.

The study sample size was not based on a formal power calculation but instead on generally acceptable participant numbers for a first-in-human study. A sample size of six healthy participants on active drug at each dose level is believed to adequately meet the study objectives, obtain initial safety and tolerability information, and estimate pharmacokinetic parameters at each dose level for an anticipated human therapeutic dose level.

## Results

### Subjects

Of 290 participants screened, 91 participants were enrolled and randomized for the study from September 22, 2017 to April 30, 2018. In the single-dose cohorts, 55 participants enrolled and were randomized across 7 cohorts (S1–S7); 41 participants received a single dose of paltusotine (n = 6 per cohort except for cohort S5 where n = 5) and 14 participants received placebo (n = 2 in each cohort) (Fig. 1). In the 20 mg cohort (S5), one participant withdrew from the study prior to dosing and was not replaced. All single-dose participants who were administered paltusotine or placebo completed the study. In the 4 multiple-dose cohorts, 36 volunteers were enrolled and randomized, and included 24 participants who received at least one dose of paltusotine and 12 who received placebo. Two multiple-dose participants discontinued the study before completion—one participant receiving paltusotine 5 mg withdrew due to a TEAE, and one participant receiving paltusotine 10 mg discontinued due to schedule conflicts. Table 1 summarizes the participants’ characteristics. The majority of the single-dose (71%) and multiple-dose participants (69%) were Caucasians. All participants were male.

**Table 1:** Demographics of participants in single- and multiple-dose groups

	SAD			MAD		
	Paltusotine	Placebo	All participants	Paltusotine	Placebo	All participants
	(n = 41)	(n = 14)	(n = 55)	(n = 24)	(n = 12)	(n = 36)
Age (years)	25.9 ± 5.4	29.1 ± 7.3	26.7 ± 6.0	26.1 ± 6.2	26.3 ± 5.2	26.2 ± 5.8
Male	41 (100%)	14 (100%)	55 (100%)	24 (100%)	12 (100%)	36 (100%)
Weight (kg)	75.4 ± 9.8	80.8 ± 15.1	76.7 ± 11.5	75.5 ± 11.5	71.6 ± 10.7	74.2 ± 11.3
BMI (kg/m^2^)	23.6 ± 2.8	24.2 ± 3.1	23.8 ± 2.9	23.5 ± 2.8	22.8 ± 2.3	23.3 ± 2.6

Data are presented as mean ± standard deviation, and number (%)

*BMI* body mass index, *SAD* single ascending dose, *MAD* multiple ascending dose

### Safety and tolerability

The safety population included all 55 participants in the single-dose cohorts and 36 participants in the multiple-dose cohorts who received study drug. The most common TEAEs in single-dose paltusotine-treated participants were headaches (n = 5) (Table 2). In multiple-dose paltusotine-treated participants, the most common TEAEs were abdominal pain (n = 4), diarrhea (n = 4), and headache (n = 3) (Table 3). Most of the TEAEs occurred at a dose level of 10 mg or higher (64% of TEAEs in single-dose cohorts and 78% of TEAEs in multiple-dose cohorts).

**Table 2:** Treatment-emergent adverse events in all participants with single-dosing—safety population

	Paltusotine (N = 41) n (%)	Placebo (N = 14) n (%)
Participants with at least 1 TEAE	28 (68.3%)	4 (28.6%)
Headache	5 (12.2%)	0 (0.0%)
Abdominal distension	2 (4.9%)	0 (0.0%)
Abdominal pain	2 (4.9%)	0 (0.0%)
Abdominal pain upper	2 (4.9%)	0 (0.0%)
Nausea	2 (4.9%)	0 (0.0%)
Hypoesthesia	2 (4.9%)	0 (0.0%)
Tension headache	2 (4.9%)	0 (0.0%)
Fatigue	2 (4.9%)	0 (0.0%)
Ventricular tachycardia (non-sustained)^a^	2 (4.9%)	0 (0.0%)
Back pain	2 (4.9%)	0 (0.0%)

Data are n (%) of participants who experience TEAEs in each dose group

TEAE = treatment-emergent adverse events

TEAEs occurring more than once in the safety population are shown

^a^Documented non-sustained ventricular tachycardia (NSVT) episodes also occurred prior to dosing (non-treatment emergent) and are described in Results section

**Table 3:** Treatment-emergent adverse events in all participants with multiple-dosing—safety population

	Paltusotine (N = 24) n (%)	Placebo (N = 12) n (%)
Participants with at least 1 TEAE	17 (70.8%)	7 (58.3%)
Abdominal pain	4 (16.7%)	0 (0.0%)
Diarrhea	4 (16.7%)	0 (0.0%)
Headache	3 (12.5%)	0 (0.0%)
Ventricular tachycardia (non-sustained)^a^	2 (8.3%)	0 (0.0%)
Lipase/pancreatic enzymes increased	2 (8.3%)	0 (0.0%)
Hyperglycemia^b^	2 (8.3%)	0 (0.0%)
Upper respiratory tract infection	2 (8.3%)	0 (0.0%)

Data are n (%) of participants who experience TEAEs in each dose group and number of TEAE occurrences

*TEAE* treatment-emergent adverse events

TEAEs occurring more than once in the safety population are shown

^a^Documented non-sustained ventricular tachycardia (NSVT) episodes also occurred prior to dosing (non-treatment emergent) and are described in Results section

^b^Both events were asymptomatic, occurred 1 h post-oral glucose load (peak glucose values 220 and 223 mg/dL), and resolved by 2 h post-load during the scheduled oral glucose tolerance test following the completion of 10 days of paltusotine dosing

No life-threatening events or deaths occurred. During protocol specified Holter and telemetry monitoring, eight subjects were noted incidentally to experience brief, non-sustained ventricular tachycardia (NSVT), an overall frequency consistent with those reported in healthy populations undergoing continuous cardiac monitoring [15, 16]. Of these eight subjects, four subjects had NSVT following dosing with paltusotine, while four subjects had NSVT prior to dosing. Only NSVT episodes occurring after paltusotine dosing (two single dose and two multiple dose participants) were reported as treatment emergent adverse events (see Tables 2 and 3). All NSVT events were assessed as unlikely related or unrelated to study drug. One of the NSVT episodes was reported as a serious adverse event occurring during the single-dose phase in a 29-year-old male participant. In this case, NSVT was of 10 s duration and associated with transient palpitations and lightheadedness. The event occurred approximately 13 h after receiving a single dose of paltusotine 1.25 mg oral solution. This event was determined to be unlikely related to paltusotine.

Two subjects were reported to have adverse events associated with amylase or lipase elevations. One of these (cohort M3, 20 mg) discontinued paltusotine after Day 5 of dosing due to elevated amylase and lipase peaks 2 and 3.9 times the upper limit of normal, respectively. The amylase and lipase elevations resolved after study drug discontinuation and there were no clinical sequelae associated with this event. The second subject (cohort M4, 30 mg) experienced a lipase elevation 1.7 times the upper limit of normal with normal amylase 24 h after the last (tenth dose) of paltusotine. The lipase elevation resolved without sequelae upon follow-up. Amylase/lipase elevations are a known class effect of somatostatin receptor ligands [17, 18].

Overall, vital signs measurements, clinical chemistry, and hematological assessments showed no evidence for clinically significant changes. There were no clinically meaningful changes in heart rate, ECG morphology, atrioventricular conduction (as measured by PR duration), cardiac depolarization (as measured by QRS interval), or cardiac repolarization (as measured by QTcF interval). There were no consistent trends of clinical relevance in TSH, ACTH, or prolactin levels observed with repeated administration regardless of treatment assignment or paltusotine dose level. Compared to baseline, there was a small but significant increase in glucose AUC_0-3_ upon 75 g oral glucose challenge at the highest multiple dose of 30 mg (3.2 h·mmol/L; 95% CI 1.8–4.6) compared to a smaller and not significant change in placebo (1.5 h·mmol/L; 95% CI − 1.5–4.6). Fasting plasma glucose was not significantly changed.

### Single ascending dose pharmacokinetics and pharmacodynamics

In the single-dose cohorts receiving the oral solution in a fasted state, paltusotine was rapidly absorbed, reaching median peak concentrations at approximately 1.3 to 2.2 h (Tmax), followed by an apparent terminal elimination (t1/2) in the range of 22 to 29 h. Exposure as measured by Cmax and AUC0-inf increased in a manner proportional to the dose in the dose range of 1.25 to 20 mg (Table 4).

**Table 4:** Pharmacokinetic parameters of single-dose paltusotine

Dose (mg)	Formulation	N	C_max_ (ng/mL)	T_max_ (h)	AUC_0-inf_	t_1/2_ (h)
1.25	Oral solution	6	9.81 ± 1.56	1.3 ± 0.44	122 ± 25.7	22 ± 5.5
2.5	Oral solution	6	22.7 ± 4.74	1.6 ± 0.80	320 ± 66.3	29 ± 9.0
5	Oral solution	6	48.1 ± 7.05	1.3 ± 0.41	590 ± 93.7	25 ± 9.4
10	Oral solution	6	95.2 ± 19.3	2.2 ± 1.3	1650 ± 464	29 ± 4.3
20	Oral solution	6	167 ± 29.1	1.9 ± 1.0	2960 ± 963	29 ± 5.0
40	Capsules	6	185 ± 118	3.4 ± 1.1	2650 ± 1200	27 ± 3.1
60	Capsules	6	154 ± 77.6	3.0 ± 1.2	2300 ± 1060	25 ± 2.1

Data are presented as mean ± standard deviation

*SAD* single ascending dose, *C*_*max*_ peak plasma concentration, *T*_*max*_ time of peak concentration, *AUC*_*0-inf*_ area under the plasma concentration time curve from 0 h to infinity, *t*_*1/2*_ apparent terminal elimination half-life

**Fig. 2:**
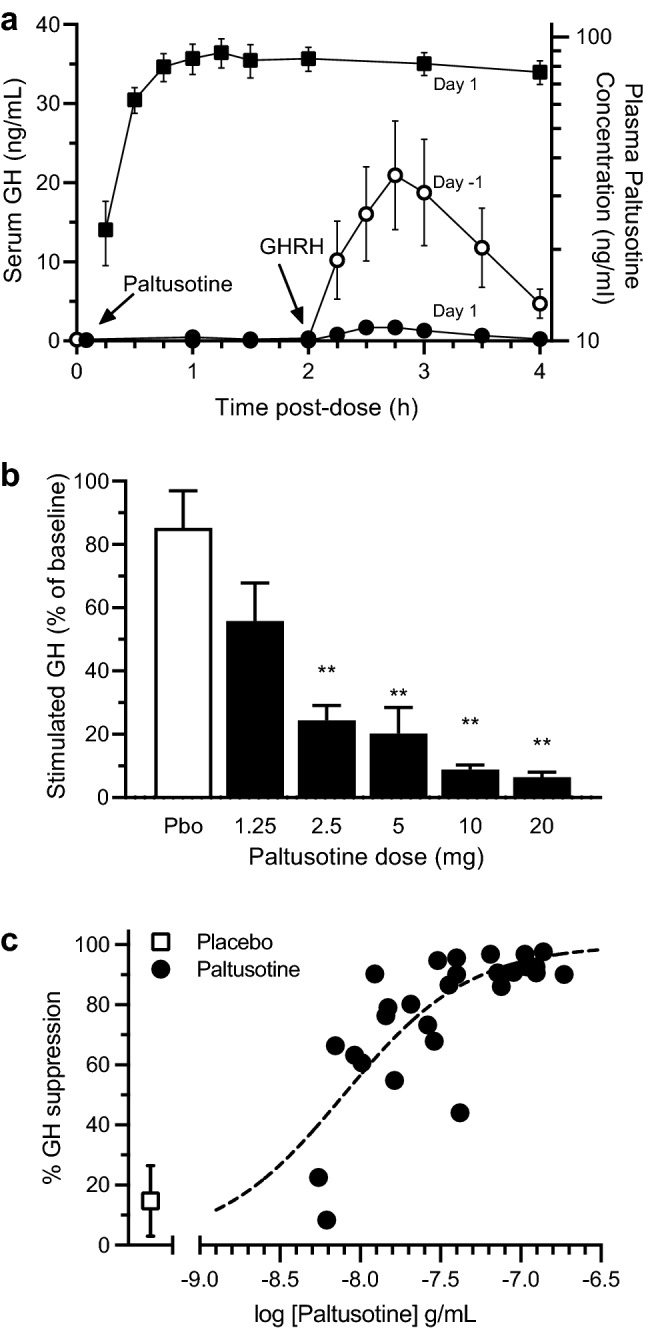
Growth hormone suppression with single-dose paltusotine. Data shown in panels a and b are mean ± SEM. **P < 0.001. *GH* growth hormone, *GHRH* growth hormone-releasing hormone, *AUC*_*0-2*_ area under the serum concentration time curve from 0 to 2 h; EC_80_ = 80% GH suppression. On Day 1, GHRH was administered 2 h post paltusotine dose. On Day -1, GHRH was administered at approximately the same time of the day as Day 1. **a** Plasma GH concentrations upon GHRH challenge on Day -1, and Day 1 on paltusotine 10 mg. GH levels were measured at the same time of the day after a GHRH challenge on Day -1 (open circles) and on Day 1 (closed circles). Plasma paltusotine concentrations are shown in black squares. Mean peak GH response on Day 1 and Day 1 were 21 ng/mL and 1.7 ng/mL, respectively. **b** Mean GHRH-stimulated GH AUC_0-2_ percent of baseline (Day 1/Day 1) by each dose group. P values were calculated for each dose of paltusotine compared to placebo using ANOVA with Dunnett multiple comparison adjustment. **c**. Suppression of GH AUC_0-2_ post GHRH (Day-1–Day 1)/Day-1 as a function of paltusotine concentration (average of 2-, 3-, and 4-h post-dose)

The effect of paltusotine plasma concentrations on GH secretion was assessed using a GHRH challenge. Administration of GHRH alone on Day -1 resulted in a rise in serum GH with a mean peak of 21 ng/mL at approximately 1 h, which then declined rapidly and returned to near-baseline values by approximately 2 h (Fig. 2a). On the following day (Day 1) paltusotine was administered 2 h prior to GHRH challenge. The results are illustrated for the 10 mg dose in Fig. 2a and summarized for all doses including placebo in Fig. 2b. Administration of 10 mg paltusotine reduced the mean peak GH concentration to 1.7 ng/mL and suppressed the stimulated AUC_0-2 h_ by 91%. Dose-dependent suppression of stimulated GH AUC_0-2 h_ was observed as the dose was increased from 1.25 to 20 mg. Minimal changes in GH were observed in participants receiving placebo between Day -1 and Day 1. Near-maximal inhibition was observed at the 10 mg dose (91% suppression of stimulated GH AUC_0-2 h_ compared to 15% suppression with placebo; p < 0.0001 for comparison with placebo) (Fig. 2b). Mean paltusotine concentration during 0–2 h post-GHRH challenge in the dose range 1.25 mg to 20 mg and resulting %GH suppression was modeled to derive a concentration–response relationship (Fig. 2c). The paltusotine concentrations needed to achieve 50% and 80% GH suppression (EC_50_ and EC_80_) were estimated to be approximately 7.9 and 29 ng/mL, respectively. Serum IGF-1 measured after a single 1.25 to 20 mg dose of paltusotine exhibited little to no change compared to Day -1 or placebo (data not shown).

### Relative bioavailability of the capsule formulation and food effect

Relative to the oral solution, mean Cmax and AUC0-inf of the capsule formulation under fasted conditions were modestly lower (geometric mean ratios of 83% and 69%, respectively), suggesting that the capsules were a suitable formulation for multiple dosing. A small loss of relative bioavailability with a solid oral dose compared to an oral solution is expected and is likely due to the time taken for disintegration and dissolution of the capsules. Compared to the fasted state, capsules taken with a standard high-fat, high-calorie meal demonstrated a markedly lower Cmax and AUC0-inf (geometric mean ratios of 14% and 17%, respectively), longer Tmax (3.0 h compared to 2.1 h), but similar t1/2 (data not shown). Single paltusotine doses of 40 mg and 60 mg were administered in the capsule formulation. As the dose was increased from 40 to 60 mg, there was no further increase in Cmax and AUC0-inf suggesting a loss of dose proportionality in this dose range. Therefore, doses higher than 60 mg were not evaluated.

### Multiple ascending dose pharmacokinetics and pharmacodynamics

**Fig. 3:**
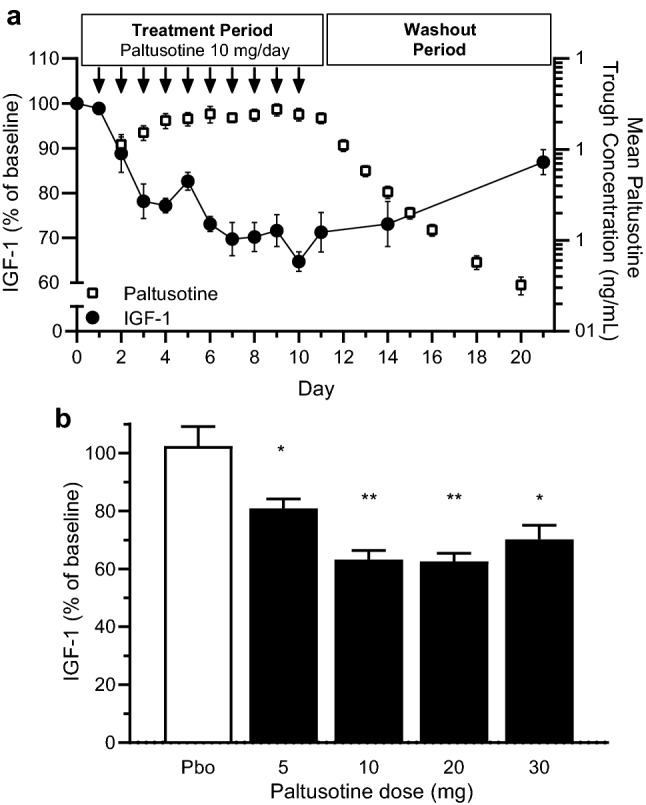
Suppression of IGF-1 with repeated administration of paltusotine. Data shown are mean ± SEM. *P < 0.05; **P < 0.001. *IGF-1* insulin-like growth factor-1; GHRH = growth hormone-releasing hormone. **a** Plasma paltusotine (open squares) and serum IGF-1 (closed circles) concentrations at 10 mg multiple dose from baseline (Day 1) to 10–11 days post-dose (Days 20–21). For this dose group, IGF-1 samples were obtained pre-dose, 6 and 12 h post-dose on Days 1, 1 and 10; pre-dose and 12 h post-dose on Days 2–9; and a single sample at approximately the same time as dosing on Days 11, 14, and 21. Arrows represent days on which paltusotine was administered (Days 1 to 10). Paltusotine concentrations are trough plasma concentrations 24 h post-dose on Days 2 to 11 and measured concentrations on Day 12 to 20 after the last dose on Day 10. Baseline IGF-1 was the average of all pre-first dose values. For each of the subsequent days, during the treatment period, each day’s pre-dose IGF-1 value was averaged with the previous day’s value (e.g., Day 2 pre-dose value was averaged with Day 1 post-dose value). **b** Dose–response for IGF-1 suppression relative to baseline with multiple-dose paltusotine. Values are IGF-1 percent of baseline 12 h post-dose on Day 7 (5 mg dose group) and on Day 10 (10 mg, 20 mg, 30 mg dose groups). Baseline was the average of Day -1 and Day 1 pre-dose IGF-1 results. P-values were calculated for each dose of paltusotine compared to placebo using ANOVA with Dunnett multiple comparison adjustment

Multiple dosing with the capsule formulation over a dose range of 5 to 20 mg demonstrated a dose-proportional increase in plasma paltusotine Cmax and AUC0-24 on Day Last. Increases in paltusotine exposure were observed between the 20 mg and 30 mg doses; however, they were less than dose-proportional (Table 5). The steady-state trough concentrations also rose less than dose proportionally. Based on a comparison of trough plasma concentrations (Fig. 3a), paltusotine achieved steady state in 3 to 5 days following once-daily dosing. Mean t1/2 with daily administration of paltusotine at 5 mg was 34 h compared to 42–50 h for the 10–30 mg dose range. However, the lower observed mean t1/2 at the 5 mg dose level (34 h) is likely due to a shorter 144 h sampling period after the last dose compared to a 240 h sampling period for the 10 mg to 30 mg doses. Accumulation factor calculated as a ratio of steady-state AUC to AUC_0-24_ on Day 1 was variable (likely due to the variability of the capsule formulation on Day 1) and ranged from 1.5- to 3.6-fold.

**Table 5:** Pharmacokinetic parameters of paltusotine with multiple dosing

Dose (mg)	N	Day	C_max_ (ng/mL)	T_max_ (h)	AUC_0-24_ (h × ng/mL)	C_trough_ (ng/mL)	t_1/2_ (h)
5	6	1	16.8 ± 7.22	1.2 ± 0.1	167 ± 73.4	Not applicable	
		7	35.5 ± 12.0	2.1 ± 0.6	421 ± 163	10.8 ± 5.11	34 ± 4.7
10	6	1	78.7 ± 45.3	1.8 ± 0.9	661 ± 340	Not applicable	
		10	75.5 ± 37.4	3.0 ± 1.8	956 ± 371	22.2 ± 7.38	42 ± 5.0
20	5	1	88.7 ± 43.3	2.4 ± 0.8	811 ± 409	Not applicable	
		10	132 ± 79.2	1.6 ± 0.4	1560 ± 733	37.9 ± 11.4	50 ± 6.6
30	5	1	78.2 ± 60.7	1.4 ± 0.9	578 ± 411	Not applicable	
		10	154 ± 102	4.5 ± 2.1	2080 ± 1220	51.1 ± 32.0	46 ± 7.4

Data are presented as mean ± standard deviation

For the 5 mg cohort, pharmacokinetic samples were collected on Day 1 (pre-dose, every 15 min up to 90 min post-dose, then at 2, 3, 4, 6, 8, 10, 12, and 18 h post-dose), Days 2–6 (pre-dose and 2 h post-dose), Day 7 (same time points as Day 1, followed by samples at 18, 24, 48, 72, 96, 120, and 144 h post-dose). For all other multiple-dose cohorts, pharmacokinetic samples were collected on Day 1 (pre-dose, every 15 min up to 90 min post-dose, then at 2, 3, 4, 6, 8, 10, 12, and 18 h post-dose), Days 2–6 (pre-dose and 2 h post-dose), Day 10 (same time points as Day 1, followed by samples at 18, 24, 48, 72, 96, 120, 144, 192, and 240 h post-dose)

*AUC*_*0-24*_ area under the plasma concentration time curve from 0 to 24 h; *C*_*max*_ peak plasma concentration; *t*_*1/2*_ apparent terminal elimination half-life

The effect of paltusotine on serum IGF-1 concentrations was evaluated in the multiple-dose cohorts during repeated daily administration of paltusotine. As illustrated for the 10 mg cohort in Fig. 3a, as paltusotine concentrations reached steady state after 3 to 5 days, serum IGF-1 concentrations declined and reached near-maximum suppression in approximately 7 days. Following the final dose of paltusotine on Day 10, IGF-1 remained suppressed for an additional 4 days and began to approach normal levels on Day 20. Mean IGF-1 concentrations were significantly reduced compared to placebo at all dose levels (Fig. 3b). At the end of the multiple-dosing period, IGF-1 increased by 2.4% in the placebo group, in contrast to a 19% suppression with paltusotine 5 mg on Day 7, and a 37%, 37%, and 30% suppression with 10 mg, 20 mg, and 30 mg, respectively, on Day 10. The 30% suppression of IGF-1 at the 30 mg dose compared to maximal 37% suppression at the lower 10 and 20 mg doses was not considered meaningful and attributed to variability due to a small sample size (n = 5–6) in each cohort.

## Discussion

This first-in-human study was designed to evaluate the safety, PK, and PD of paltusotine, a novel nonpeptide oral SST2 agonist. Single-dose administration of paltusotine in healthy volunteers was well tolerated. Repeated daily administration of paltusotine capsules in healthy volunteers over 7 days (5 mg) and 10 days (10 mg, 20 mg, and 30 mg) was also well tolerated. TEAEs included headache and various non-specific gastrointestinal symptoms, consistent with the known side effects of SRL therapy. Elevations in pancreatic enzymes have previously been reported in subjects treated with SRL treatments [17–20]. Modest postprandial hyperglycemia demonstrated via oral glucose tolerance, without changes in fasting glucose or HbA1c, has also been previously described with this pharmacologic class [21]. This is in contrast to the less selective pasireotide which has potent activity for SST5 receptor and is causally associated with decrease in insulin secretion and hyperglycemia [18, 22]. The most common cardiac disorder associated with SRLs is bradycardia [20, 23]. In this study, clinically meaningful changes in heart rate were not apparent in healthy volunteers given up to 30 mg repeated daily administration of paltusotine. A variety of cardiac rhythm disorders have been reported with SRLs including ventricular arrhythmias [20, 24]. In this study, continuous Holter recordings and telemetry showed the sporadic occurrence of NSVT with a similar prevalence during both pre-dose and post-dose cardiac monitoring. Studies conducted in healthy volunteers have demonstrated frequencies of NSVT in 2–5% of healthy individuals; the prevalence of NSVT in this trial therefore is within the range for healthy subjects [15, 16]. There was no evidence of paltusotine-related changes in ECG morphology, ECG parameters, atrioventricular block, or supraventricular tachyarrhythmias. Placebo-controlled evaluation of safety and efficacy of paltusotine in Phase 3 trials is underway.

Paltusotine was rapidly absorbed after oral administration. The t_1/2_ of paltusotine ranged from 22 to 34 h with pharmacokinetic sampling up to 144 h post-dose (i.e., in single-dose cohorts and the 5 mg multiple-dose cohort). When sampled up to 240 h after the last dose, the t_1/2_ of multiple-dose paltusotine ranged from 42 to 50 h. The longer terminal elimination t_1/2_ with multiple dosing is likely due to the longer sampling time [25]. After repeated administration, trough concentrations of paltusotine do not increase beyond 4 days (Fig. 3a). Assuming that steady state is achieved in 4 to 5 t_1/2_’s, the mean effective t_1/2_ of paltusotine is likely closer to that estimated after a single dose in the range of 22 to 34 h. Consistent with the estimated single-dose t_1/2_ of paltusotine, in a separate study in which paltusotine in solution was administered intravenously and orally [26], mean absolute oral bioavailability of paltusotine oral solution was 70% and the t_1/2_ after intravenous administration was 29 h (with sampling up to 144 h). Paltusotine’s pharmacokinetic profile supports a once-daily dosing strategy in future clinical trials.

With multiple dosing of the capsule formulation, Day-10 trough concentrations, C_max_, and AUC_0-24_ increased dose proportionally from 5 to 20 mg, but were less than dose-proportional when the dose was increased from 20 to 30 mg. Also notable is that there was no further increase in C_max_ or AUC_0-24_ when the dose was increased from 40 to 60 mg in the single-dose cohorts. This suggests a saturation of absorption processes as the dose is increased above 40 mg. While the exact reason for the saturation of absorption is unknown, it is likely due to the low solubility of paltusotine in higher pH environments. A large negative food effect on paltusotine pharmacokinetics was also observed. A high-fat, high-calorie meal before intake of the 10 mg capsule markedly decreased drug absorption, as demonstrated by a more than 7-fold decrease in C_max_ and AUC_0-24_. The food effect would also be consistent with decreased solubility of paltusotine in the higher pH environment of the fed state. These data informed the design of the studies in acromegaly patients. Specifically, a maximum dose of 40 mg was selected because of saturation of absorption and patients were instructed to take the drug on an empty stomach and wait for at least 2 h before eating. Additionally, these data provide a rationale to develop an improved formulation of paltusotine that could allow dosing higher than 40 mg and reduce the impact of food effect. Recently, data from a new spray-dried dispersion tablet formulation of paltusotine was reported. This new formulation exhibits dose proportionality at least up to an 80 mg dose, is less sensitive to effects of proton pump inhibitors, and offers a shorter post-dose (1 h) fasting requirement [27].

Paltusotine administration led to robust inhibition of GHRH-stimulated GH secretion and suppression of circulating IGF-1 levels. Paltusotine suppression of stimulated GH secretion was dose-dependent, reaching near-maximal suppression at the 10 mg dose (Fig. 2b). Prior pharmacodynamic studies of peptide somatostatin receptor ligands (octreotide, lanreotide, and pasireotide) in healthy volunteers have shown similar suppression of GHRH-stimulated GH secretion [28–30]. Repeated dosing of paltusotine led to a reduction in IGF-1 levels, with near-maximal suppression achieved at the 10 mg dose level (Fig. 3b). As trough paltusotine concentrations reached steady state, serum IGF-1 concentrations began to decline (Fig. 3c). Maximal IGF-1 suppression reached its nadir in approximately 7 days. Unlike the rapid suppression of GH, IGF-1 levels decrease gradually and require 4 to 10 days of exposure to SST agonists to produce an observable effect [31]. The gradual decrease of IGF-1 is likely due to its long t_1/2_ (approximately 15 h), which is due to high affinity IGF-1 binding proteins in plasma [10]. The maximal degree of IGF-1 suppression (up to 37%) by paltusotine was similar to that observed for octreotide and lanreotide in previously reported healthy volunteer studies [17, 31]. The distinction however is that paltusotine achieves a steady state within 7 days whereas depot injections require several weeks or months to reach steady state [17, 32] and dose optimization with paltusotine is possible in much shorter time. After the final paltusotine dose, IGF-1 remained suppressed for several days but began to recover as paltusotine plasma concentrations declined. Analogous to the observation that it takes several days to achieve the nadir of IGF-1 suppression with once-a-day dosing of paltusotine, it takes several days for IGF-1 to recover back to baseline levels upon paltusotine’s withdrawal.

Paltusotine’s EC_50_ and EC_80_ for suppression of stimulated GH secretion were estimated to be 7.9 ng/mL and 29 ng/mL, respectively, based on exposure–response modeling shown in Fig. 2c. While EC_50_ is traditionally used as a measure of pharmacological potency, the EC_80_ has utility in the dose selection for the treatment of acromegaly and neuroendocrine tumors. In clinical practice, acromegaly is pharmacologically treated by dose titration of somatostatin agonists where the goal is to maximize the suppression of GH secretion at the pituitary somatotrophs, which results in suppression of serum IGF-1. There is a high degree of variability in this response presumably due to inter-individual differences in the size and responsiveness of the residual tumor, the baseline (untreated) levels of serum GH and resulting serum IGF-1, expression of SST2 receptors in the tumor cells, the contribution of the GH receptor toward total IGF-1 secretion from the liver, and other unknown factors [33–35]. This complexity leads to individualized treatment algorithms that are driven by biochemical response to the SRLs and its ability to normalize IGF-1. Despite maximal suppression of GH axis achievable with SRLs, IGF-1 is not normalized in a large percentage of patients and most patients are ultimately titrated to the highest approved dose of octreotide or lanreotide [8]. Therefore, the goal for treatment of acromegaly is to identify a dose range that results in maximal suppression of GH and IGF-1 by achieving trough concentrations that are at the EC_80_ or higher. Similarly, the goal for the treatment of GEPNETs is to maximize agonist activity at tumors expressing somatostatin receptors, and as such patients are often escalated to higher than approved doses of octreotide or lanreotide to mitigate tumor progression and/or to control carcinoid syndrome symptoms [36, 37]. More plainly, plasma drug concentrations that are sub-maximal in their ability to suppress GH (e.g., EC_50_) are unlikely to have clinical utility. This is consistent with the observation that, in healthy volunteers, the mean steady state trough concentration that resulted in near-maximal suppression of IGF-1 was 22.2 ng/mL (observed at the 10 mg dose), which was similar to the EC_80_ of stimulated GH secretion (29 ng/mL). Acromegaly patients may require similar or higher steady-state trough paltusotine concentrations depending on the activity of the GH-secreting tumor. Doses necessary to achieve a target effective trough concentration in acromegaly patients may also be higher because of factors such as higher body weight and/or differences in body composition. Nevertheless, this healthy volunteer data provides a valuable approximation of a starting dose in acromegaly patients such that patient studies could be initiated without the need for extensive dose exploration. In addition, these data provide information on time to steady state of the pharmacodynamic effect, time needed for complete washout of study drug and reversal of its pharmacodynamic effect, safety and tolerability of the doses to be explored in patients, and drug-class specific safety monitoring requirements, which in turn informs the design of patient studies.

Two acromegaly phase 2 trials that incorporated the information and conclusions from the Phase 1 trial have recently been completed: ACROBAT EDGE, which evaluated the safety and efficacy of paltusotine in acromegaly patients whose IGF-1 concentrations were reduced but did not achieve normalization by octreotide or lanreotide depots (NCT0389656), and ACROBAT EVOLVE, which evaluated the safety and efficacy of paltusotine in acromegaly patients whose IGF-1 concentrations were reduced and achieved normalization by octreotide or lanreotide (NCT03792555) [38, 39]. Full results from these studies will be reported elsewhere. Similarly, future clinical trials in carcinoid syndrome and neuroendocrine tumors will be informed by the results of the current healthy volunteer study.

Peptide, parenterally administered drugs are available for several validated therapeutic targets for treating endocrine diseases. Medicinal chemistry combined with understanding of drug-like properties necessary to make an orally bioavailable small molecule enables rapid discovery and development of therapeutics that can replace inconvenient parenteral therapies. For example, discovery and development of elagolix for the treatment of endometriosis and uterine fibroids and that of several angiotensin II receptor blockers have used similar approaches [40–42]. The demonstration of GH and IGF-1 suppression in healthy volunteers by the orally bioavailable small molecule drug paltusotine is another example of an efficient drug discovery and development platform.

## Limitations

Although we attempted to enroll both male and female participants, all participants were men, the great majority of whom were under 30 years of age.

## Conclusion

Data from this phase 1 clinical trial in healthy volunteers demonstrate that paltusotine is generally safe and well tolerated. Robust reduction of stimulated GH and IGF-1 was observed with maximal suppression at 10 mg daily, and similar in magnitude to that observed in healthy volunteers receiving currently approved peptide somatostatin analogs. The sustained suppression of IGF-1 observed after repeated daily dosing of paltusotine combined with an approximate t_1/2_ of 1 to 2 days supports a once-daily oral dosing strategy. Collectively, the pharmacokinetic profile, GH and IGF-1 suppression, and safety data support further clinical development of paltusotine in patients with acromegaly and neuroendocrine tumors.

## Data Availability

Some or all datasets generated during and/or analyzed during the current study are not publicly available but are available from the corresponding author on reasonable request.
